# Variability of Urinary Phthalate Metabolite and Bisphenol A Concentrations before and during Pregnancy

**DOI:** 10.1289/ehp.1104139

**Published:** 2012-01-19

**Authors:** Joe M. Braun, Kristen W. Smith, Paige L. Williams, Antonia M. Calafat, Katharine Berry, Shelley Ehrlich, Russ Hauser

**Affiliations:** 1 Department of Environmental Health, and; 2 Department of Biostatistics, Harvard School of Public Health, Boston Massachusetts, USA; 3 National Center for Environmental Health, Centers for Disease Control and Prevention, Atlanta, Georgia, USA; 4 Division of Reproductive Medicine, Department of Obstetrics, Gynecology, and Reproductive Biology, Brigham and Women’s Hospital, Boston, Massachusetts USA

**Keywords:** bisphenol A, endocrine disruptors, epidemiology, phthalates, pregnancy, variability

## Abstract

Background: Gestational phthalate and bisphenol A (BPA) exposure may increase the risk of adverse maternal/child health outcomes, but there are few data on the variability of urinary biomarkers before and during pregnancy.

Objective: We characterized the variability of urinary phthalate metabolite and BPA concentrations before and during pregnancy and the ability of a single spot urine sample to classify average gestational exposure.

Methods: We collected 1,001 urine samples before and during pregnancy from 137 women who were partners in couples attending a Boston fertility clinic and who had a live birth. Women provided spot urine samples before (*n* ≥ 2) and during (*n* ≥ 2) pregnancy. We measured urinary concentrations of monoethyl phthalate (MEP), mono-*n*-butyl phthalate (MBP), mono-iso-butyl phthalate, monobenzyl phthalate (MBzP), four metabolites of di-(2-ethylhexyl) phthalate (DEHP), and BPA. After adjusting for specific gravity, we characterized biomarker variability using intraclass correlation coefficients (ICCs) and conducted several surrogate category analyses to determine whether a single spot urine sample could adequately classify average gestational exposure.

Results: Absolute concentrations of phthalate metabolites and BPA were similar before and during pregnancy. Variability was higher during pregnancy than before pregnancy for BPA and MBzP, but similar during and before pregnancy for MBP, MEP, and ΣDEHP. During pregnancy, MEP (ICC = 0.50) and MBP (ICC = 0.45) were less variable than BPA (ICC = 0.12), MBzP (ICC = 0.25), and ΣDEHP metabolites (ICC = 0.08). Surrogate analyses suggested that a single spot urine sample may reasonably classify MEP and MBP concentrations during pregnancy, but more than one sample may be necessary for MBzP, DEHP, and BPA.

Conclusions: Urinary phthalate metabolites and BPA concentrations were variable before and during pregnancy, but the magnitude of variability was biomarker specific. A single spot urine sample adequately classified MBP and MEP concentrations during pregnancy. The present results may be related to unique features of the women studied, and replication in other pregnancy cohorts is recommended.

Phthalates and bisphenol A (BPA) are multifunctional compounds used in a variety of commercial and industrial products. Diethyl phthalate (DEP), di-*n*-butyl phthalate (DBP), and benzylbutyl phthalate (BzBP) can be used in personal care and consumer products to hold scent and color [National Research Council (NRC) 2008]. DBP, BzBP, and di-(2-ethylhexyl) phthalate (DEHP) can also be used in the manufacture of floorings, carpet backings, adhesives, wallpaper, and polyvinyl chloride (PVC) plastics (NRC 2008). BPA can be used in food can linings, water supply pipes, medical tubing, thermal receipts, and cigarette filters (Biedermann et al. 2010; Chapin et al. 2008; Jackson and Darnell 1985). Exposure to BPA and phthalates is nearly ubiquitous among persons in the United States (Calafat et al. 2008; Koch and Calafat 2009; Woodruff et al. 2011).

Animal studies demonstrate that gestational phthalate and BPA exposures are associated with adverse health outcomes (Chapin et al. 2008; NRC 2008). Epidemiological studies suggest that exposure to some phthalates and BPA may be associated with adverse neurodevelopmental outcomes (Braun et al. 2009; Miodovnik et al. 2011; Swan et al. 2010). However, the potentially episodic nature of exposure, combined with the short biological half-lives of these compounds raises questions about the adequacy of a single spot urine sample to classify gestational BPA and phthalate exposure. Variations in the patterns of food consumption may contribute to the within-person variability of phthalate and BPA exposure, while patterns of personal care product use and movement between environments with variable air and dust concentrations may create additional within-person variability of phthalate exposure (Adibi et al. 2008; Preau et al. 2010; Ye et al. 2011). In addition, pregnancy-related changes in xenobiotic metabolism may contribute to the variability of urinary BPA and phthalate metabolite concentrations throughout gestation. As far as we are aware, no prior studies have examined the variability of urinary BPA and phthalate metabolite concentrations in the same woman before and during pregnancy.

A better understanding of the variability of urinary phthalate and BPA biomarkers can help investigators determine the adequacy of these markers to classify phthalate and BPA exposure during critical periods of development such as pregnancy. The purpose of this study was to characterize the pattern, variability, and reproducibility of DEHP metabolites, mono-*n*-butyl (MBP), mono-iso-butyl phthalate (MiBP), mono-benzyl phthalate (MBzP), monoethyl phthalate (MEP), and BPA concentrations in serial urine samples from 137 women before and during pregnancy. In addition, we determined whether a single spot urine sample during the first, second, or third trimester could accurately classify average gestational phthalate and BPA exposure. This information will help inform exposure assessment in epidemiological studies and aid in the evaluation of human studies that use a single spot urine sample to classify these exposures.

## Methods

Women 18–45 years of age were recruited from partners seeking evaluation and treatment for infertility at the Massachusetts General Hospital (MGH) Fertility Center in Boston between November 2004 and December 2009. The present analyses were from a larger prospective open-cohort study, the Environment and Reproductive Health (EARTH) Study, which was designed to examine the relationship between environmental chemical exposures and fertility/pregnancy outcomes. The study was approved by the human studies institutional review boards of the MGH, Harvard School of Public Health and the Centers for Disease Control and Prevention (CDC). Subjects signed an informed consent form after the study procedures were explained by a research nurse and all questions were answered.

Women included in this analysis were recruited preconception (herein referred to as pre- or before pregnancy) and followed until delivery. Conception methods included natural conception, ovulation induction with timed intercourse, intrauterine insemination, or *in vitro* fertilization. Women provided spot urine samples in polypropylene containers at enrollment, on returning for subsequent clinic appointments before pregnancy, and during pregnancy (first, second, or third trimester). Enrollment urine samples were generally collected on entry into the study and before fertility treatment. Before storing samples at –80°C, urine was aliquoted and specific gravity (SG) was measured using a handheld refractometer calibrated with deionized water before each use (National Instrument Company Inc, Baltimore, MD). Samples were shipped on dry ice to the CDC for analysis.

An intrauterine pregnancy was confirmed by presence of a fetal heartbeat detected by transvaginal ultrasound. We used one of three methods to estimate the date of conception: oocyte retrieval date, which was abstracted from medical records; crown–rump length, which was measured during a fetal ultrasound between 6 and 8 weeks of gestation; or woman’s report of last menstrual period. When more than one dating method was available, priority was given to retrieval date > ultrasound > last menstrual period.

To examine variability of urinary phthalate metabolite concentration before and during pregnancy, we restricted our analyses to women who delivered a liveborn infant and provided two or more pregnancy urine samples and two or more urine samples before that pregnancy. We excluded women with fewer than two urine samples during either or both time periods.

We measured the concentration of BPA and eight phthalate metabolites including MBP, MiBP, MBzP, MEP, and the following four DEHP metabolites: mono(2-ethyl-5-carboxypentyl) phthalate (MECPP), mono(2-ethyl-5-hydroxyhexyl) phthalate (MEHHP), mono(2-ethyl-5-oxohexyl) phthalate (MEOHP), and mono(2-ethylhexyl) phthalate (MEHP), using previously described analytical chemistry methods and quality control procedures. (Silva et al. 2007; Ye et al. 2008) We limited our statistical analyses to DEHP metabolites, MBP, MiBP, MBzP, and MEP because of their high frequency of detection in the U.S. population (Silva et al. 2004; Woodruff et al. 2011) The limits of detection (LOD) for the target phthalate metabolites were in the low microgram per liter range (~ 0.1 to ~ 1 μg/L) and 0.4 μg/L for BPA. Values less than the LOD were given a value of the LOD/_√_^–^2 (Hornung and Reed 1990). We applied correction factors of 0.66 and 0.72 to the MEP and MBzP concentrations, respectively, because the analytic standards used were of inadequate purity (Calafat A, personal communication).

Because DEHP is metabolized primarily into MEHP, MEHHP, MECPP, and MEOHP, we used two summary measurements: *a*) total molar sum of all four DEHP metabolites and *b*) molar sum of three oxidative DEHP metabolites (MECPP, MEHHP, and MEOHP). We calculated the molar sum of DEHP metabolites by dividing each metabolite concentration by its molar mass and then summing the individual metabolite concentrations. We also present results separately for MEHP concentrations to facilitate comparisons with prior studies.

We accounted for urine dilution by standardizing urinary phthalate metabolite and BPA concentrations using SG. Urine dilution was adjusted using a modified and previously described formula in all of our analyses of BPA and phthalate metabolites (Duty et al. 2005; Meeker et al. 2009). We excluded samples with SG values > 1.04 (Boeniger et al. 1993). All statistical analyses were conducted using SG-standardized biomarker concentrations unless otherwise noted.

*Statistical analyses.* Descriptive analyses. We first examined the sociodemographic characteristics of participating women (means and proportions). We computed the median and 25th and 75th percentiles of SG-adjusted phthalate metabolites and BPA concentrations from the first (at enrollment) and last urine samples provided before women became pregnant and from samples provided during each trimester of pregnancy. We calculated univariate characteristics of and correlation between the within-woman geometric mean (GM) urinary phthalate metabolite and BPA concentrations for all prepregnancy and pregnancy urine samples. We also compared the difference in phthalate metabolite and BPA concentrations before and during pregnancy using a linear mixed model with log_10_-transformed phthalate metabolite or BPA concentrations as the outcome. We included an indicator variable to designate samples as being before or during pregnancy. We estimated the percent difference in pregnancy concentrations relative to prepregnancy concentrations.

Variability analyses. We conducted three analyses to characterize the variability and change in urinary phthalate metabolite and BPA concentrations before and during pregnancy. These analyses used log_10_-transformed urinary SG-adjusted phthalate metabolite and BPA concentrations because of their right-skewed distribution. First, we estimated the variability of urinary phthalate metabolite and BPA concentrations before or during pregnancy by calculating the intraclass correlation coefficient (ICC) using a random intercept-only linear mixed model. The ICC is a measure of reproducibility, calculated by dividing the between-subject variability by the sum of the between- and within-subject variability. Values range from 0, indicating no reproducibility, to 1, indicating perfect reproducibility (Rosner 2000). Next we estimated the percent change in urinary phthalate metabolite and BPA concentrations over time during the prepregnancy or pregnancy sampling frame using linear mixed models with subject-specific intercepts. For prepregnancy samples we calculated the number of weeks since enrollment by subtracting the date of enrollment from each subsequent collection date. The enrollment urine sample was set to a time of 0. For pregnancy samples, we calculated the number of weeks since conception for each urine sample by subtracting the date of conception from each urine sample collection date. We estimated the percent change in phthalate metabolite and BPA concentration with each 4-week change in time before and during pregnancy. Finally, using spaghetti plots, we graphed a random sample of urinary phthalate metabolite and BPA concentrations in 50 women before and during pregnancy as a function of time since enrollment or conception.

We evaluated the pattern and variability of urine dilution before and during pregnancy by conducting the above analyses using untransformed SG values as the outcome, because changes in urine dilution (i.e., SG) during pregnancy may partially account for changes in urinary phthalate concentrations.

Surrogate category analyses. We conducted three additional analyses to examine the rank-ordering, predictive ability, and consistency of a single urinary phthalate metabolite and BPA concentration during pregnancy among women with all three urine samples (i.e., one sample for each trimester). First, we conducted a classification analysis (Hauser et al. 2004; Mahalingaiah et al. 2008). Using the GM of the three individual trimester phthalate metabolite or BPA concentrations, we calculated tertiles of average gestational exposure of the women. We then classified women into surrogate tertiles of phthalate metabolite/BPA concentrations using their trimester-specific urine sample concentration. We categorized the women as being either in the top or bottom two tertiles of either of these measures. We then calculated the sensitivity, specificity, and positive predictive value (PPV) of the top tertile of the surrogate measure with the top tertile of the average gestational measure. The PPV is the probability of being classified as having high average gestational concentrations given a high surrogate measure. We examined trimester-specific surrogate categories to determine if the timing of sample collection influenced the predictive ability of a single spot urine sample.

Second, we examined whether surrogate categories of trimester-specific urine samples were associated with average gestational urinary phthalate metabolite or BPA concentrations of women (Meeker et al. 2005; Teitelbaum et al. 2008). Similar to the first analysis, we calculated surrogate tertiles of trimester-specific urinary phthalate metabolite/BPA concentrations (i.e., surrogate categories of low, medium, and high). We then used box plots to examine the distribution of the average gestational exposure of women (e.g., GM of all three urinary biomarker concentrations) within each of these surrogate categories. For example, we calculated the average gestational urinary BPA concentration of each woman using the values from all three trimesters. Based on the first-trimester urinary BPA concentrations for all the women, we then categorized each woman’s first-trimester urinary BPA concentrations into surrogate tertiles. We then plotted the distribution of the average gestational urinary BPA concentration of women for the first-trimester surrogate tertile variable. We then completed the same process for the second and third trimesters. If the surrogate tertile variable provides reasonable rank ordering, then we would expect to see increasing average gestational BPA concentrations across the surrogate trimester categories (i.e., increasing average gestational BPA concentration as one goes from first to second- to third-trimester surrogate categories).

Finally, we examined whether the women remained in the same tertile of exposure over the course of their pregnancy by counting the number of times (one, two, or three) her urine sample concentrations were in the same tertile for each phthalate metabolite or BPA. For instance, if all three urine samples for a woman were in the same tertile, she was assigned a count of three. We calculated separate tertiles for each trimester. All analyses were conducted with SAS version 9.2 (SAS Institute Inc., Cary, NC).

## Results

Descriptive analyses. A total of 221 women in our study had live births. For our BPA analyses, 137 women provided two or more urine samples (*n* = 636 samples) before getting pregnant and two or more urine samples (*n* = 365 samples) during pregnancy. For phthalate metabolite analyses, 113 women provided 853 samples (544 before pregnancy and 309 during pregnancy). Women in our sample were predominately white, highly educated, and a mean (± SD) of 35 ± 4.1 years of age at enrollment (Table 1). The majority (59%) of women conceived using intrauterine insemination. Before pregnancy, women provided between 2 and 13 urine samples (median, 3 samples) during a period of < 1 to 110 weeks after enrollment (median, 12 weeks). During pregnancy, a median of three samples was collected. On average, first-, second-, and third-trimester urine samples were collected at 5 (range, 3–12 weeks), 20 (range, 12–29 weeks), and 33 (range, 26–38 weeks) weeks of gestation. A median of 5 weeks elapsed between the last prepregnancy and first pregnancy urine sample collections (range, 1–40 weeks).

**Table 1:** Demographic characteristics of 137 women participating in the EARTH study included in the present analysis.

Demographic factor	n (%)
Race	
Caucasian	124 (91)
African American	2 (1)
Asian	8 (6)
Other	3 (2)
Hispanic	
No	135 (99)
Yes	2 (1)
Education	
< Bachelor’s degree	6 (5)
Bachelor’s degree	51 (40)
Graduate degree	70 (55)
Missing	10
Maternal age at enrollment (years)	
< 30	16 (12)
30 to < 35	44 (32)
35 to < 40	61 (45)
≥ 40	16 (12)
Conception method	
Natural	11 (8)
Intrauterine insemination	81 (59)
In vitro fertilization	45 (33)

Absolute concentrations of phthalate metabolites and BPA were essentially the same before and during pregnancy [Table 2; see Supplemental Material, Table 1 (http://dx.doi.org/10.1289/ehp.1104139)]. The modest differences varied by biomarkers and were highest for MEHP [percent difference = 29%; 95% confidence interval (CI): 8, 55%] and lowest for BPA (percent difference = 6%; 95% CI: –4, 17%). MEP concentrations were 19% lower during pregnancy compared with before pregnancy (95% CI: –30, –6). However, these differences were relatively small considering the variability of the analytic chemistry methods used. Correlations between concentrations before and during pregnancy were lower for the sum of DEHP metabolites (Pearson *R* = 0.33), MEHP (Pearson *R* = 0.32), and BPA (Pearson *R* = 0.39) than for MBP (Pearson *R* = 0.62), MiBP (Pearson *R* = 0.55), MBzP (Pearson *R* = 0.54), and MEP (Pearson *R* = 0.62) (Table 2). However, the correlation coefficient for MBP was attenuated (Pearson *R* = 0.45) when we excluded one woman who had the highest prepregnancy (12,860 μg/L) and pregnancy (3,491 μg/L) urinary MBP concentration.

**Table 2:** Univariate characteristics, correlations, and difference between SG-adjusted prepregnancy and pregnancy urinary phthalate metabolite and BPA concentrations.

Urine metabolite	GM prepregnancy median (25th, 75th percentile)a	GM pregnancy median (25th, 75th percentile)a	Correlationb	Percent difference between pregnancy and prepregnancy concentration (95% CI)c
ΣDEHP metabolitesd (μmol/L)		0.33 (0.20, 0.56)		0.36 (0.18, 0.69)		0.33		9 (–8, 29)
ΣDEHP oxidative metabolitese (μmol/L)		0.30 (0.18, 0.52)		0.31 (0.17, 0.66)		0.34		8 (–9, 28)
MEHP (μg/L)	5.0 (2.8, 7.7)	5.9 (3.2, 13)	0.32	29 (8, 55)
MBP (μg/L)	14 (9.0, 22)	16 (11, 24)	0.62	25 (11, 41)
MiBP (μg/L)	5.4 (3.2, 8.9)	5.9 (4.0, 9.0)	0.55	9 (–4, 23)
MBzP (μg/L)f	3.2 (1.8, 5.2)	3.7 (2.0, 6.8)	0.54	22 (6, 40)
MEP (μg/L)f	61 (37, 145)	55 (24, 116)	0.62	–19 (–30, –6)
BPA (μg/L)		1.5 (1.1, 2.1)		1.5 (1.1, 2.3)		0.39		6 (–4, 17)
aMedian and percentiles of the within-woman GM of urinary phthalate metabolite or BPA concentrations in two or more samples obtained before pregnancy and during pregnancy. bPearson correlation coefficients between the GM of prepregnancy and pregnancy urinary phthalate metabolite or BPA concentrations (log10 transformed). cPercent difference in pregnancy versus prepregnancy urinary phthalate metabolite or BPA concentration; estimated with a linear mixed model with an indicator variable for pregnancy or prepregnancy (referent). dΣDEHP metabolites: mono(2-ethyl-5-carboxypentyl) phthalate (MECPP), mono(2-ethyl-5-hydroxyhexyl) phthalate (MEHHP), mono(2-ethyl-5-oxohexyl) phthalate (MEOHP), and mono(2-ethylhexyl) phthalate (MEHP). eΣDEHP oxidative metabolites: mono(2-ethyl-5-carboxypentyl) phthalate (MECPP), mono(2-ethyl-5-hydroxyhexyl) phthalate (MEHHP), and mono(2-ethyl-5-oxohexyl) phthalate (MEOHP). fA correction factor of 0.66 and 0.72 was applied to the MEP and MBzP concentrations, respectively.								

Variability analysis. Before pregnancy, the concentrations of DEHP metabolites, MBzP, BPA, and SG exhibited substantial within-woman variation, as evidenced by relatively low ICCs (≤ 0.35) (Table 3). Graphical examination of urinary phthalate metabolite concentrations for individual women over time supported this [see Supplemental Material, Figure 1 (http://dx.doi.org/10.1289/ehp.1104139)]. Concentrations of MBP (ICC = 0.40) and MEP (ICC = 0.56) were less variable, but some women showed variations of an order of magnitude in urinary phthalate metabolite concentration over time (Table 3; see Supplemental Material, Figure 1). Most of the urinary phthalate metabolite concentrations exhibited similar variability both before and during pregnancy (Table 3). MBzP and BPA concentrations were more variable during pregnancy than before pregnancy. We also estimated the variability of MECPP before and during pregnancy to determine whether a DEHP metabolite with a longer half-life had less variability than other DEHP metabolites (Koch et al. 2006). Similar to the other two summary DEHP metabolite measures, the variability of urinary MECPP concentrations was similar both before (ICC = 0.19) and during (ICC = 0.14) pregnancy.

**Table 3:** Intraclass correlation coefficients for and percent change (per 4 weeks) in SG–adjusted urinary phthalate metabolite/BPA concentrations and SG before and during pregnancy.

Before pregnancy			During pregnancy		
Urinary metabolite	ICCa	Percent change per 4 weeks (95% CI)b	ICCa	Percent change per 4 weeks (95% CI)b
ΣDEHP metabolitesc (μmol/L)		0.11		–1 (–3, 2)		0.08		–8 (–13, –4)
ΣDEHP oxidative metabolitesd (μmol/L)		0.12		–1 (–3, 1)		0.09		–8 (–13, –4)
MEHP (μg/L)		0.11		0 (–2, 3)		0.08		–13 (–17, –8)
MBP (μg/L)		0.40		0 (–1, 2)		0.45		–1 (–4, 3)
MiBP (μg/L)		0.36		0 (–2, 2)		0.38		3 (0, 6)
MBzP (μg/L)e		0.35		2 (0, 4)		0.25		–1 (–4, 4)
MEP (μg/L)e		0.56		–2 (–4, 0)		0.50		3 (–1, 7)
BPA (μg/L)		0.23		0 (–1, 1)		0.12		–1 (–4, 2)
SG		0.27		0.0000 (–0.0001, 0.0002)		0.37		–0.0002 (–0.0004, 0.000)
aLog10-transformed metabolite concentrations (except SG) are the outcome in a linear mixed model with a random intercept and the time in weeks since enrollment or conception as the predictor. bLog10-transformed metabolite concentrations (except SG) are the outcome in a linear mixed model with a random intercept. cΣDEHP metabolites: mono(2-ethyl-5-carboxypentyl) phthalate (MECPP), mono(2-ethyl-5-hydroxyhexyl) phthalate (MEHHP), mono(2-ethyl-5-oxohexyl) phthalate (MEOHP), and mono(2-ethylhexyl) phthalate (MEHP). dΣDEHP oxidative metabolites: mono(2-ethyl-5-carboxypentyl) phthalate (MECPP), mono(2-ethyl-5-hydroxyhexyl) phthalate (MEHHP), and mono(2-ethyl-5-oxohexyl) phthalate (MEOHP). eA correction factor of 0.66 and 0.72 was applied to the MEP and MBzP concentrations, respectively.								

**Figure 1:**
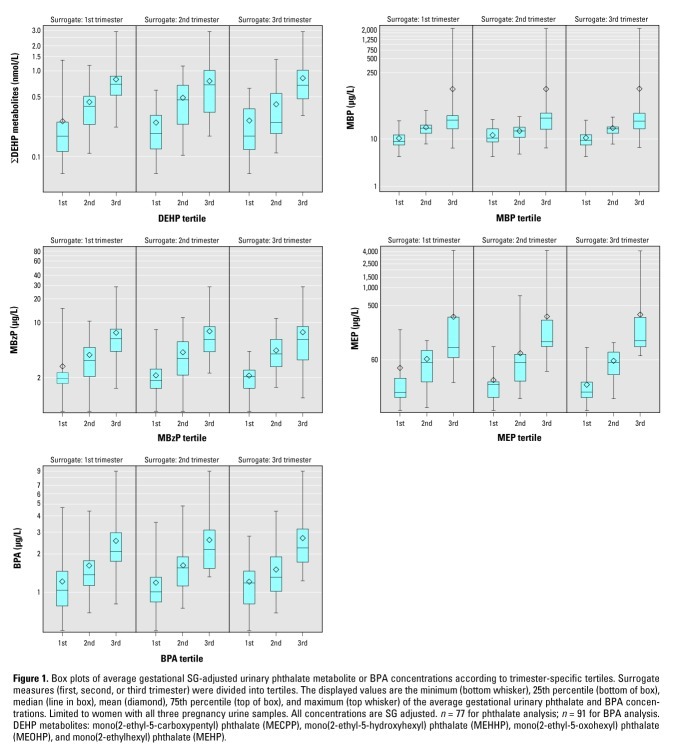
Box plots of average gestational SG-adjusted urinary phthalate metabolite or BPA concentrations according to trimester-specific tertiles. Surrogate measures (first, second, or third trimester) were divided into tertiles. The displayed values are the minimum (bottom whisker), 25th percentile (bottom of box), median (line in box), mean (diamond), 75th percentile (top of box), and maximum (top whisker) of the average gestational urinary phthalate and BPA concentrations. Limited to women with all three pregnancy urine samples. All concentrations are SG adjusted. Tertile cut points are available in Supplemental Table 1 (http://dx.doi.org/10.1289/ehp.1104139). *n* = 77 for phthalate analysis; *n* = 91 for BPA analysis. DEHP metabolites: mono(2-ethyl-5-carboxypentyl) phthalate (MECPP), mono(2-ethyl-5-hydroxyhexyl) phthalate (MEHHP), mono(2-ethyl-5-oxohexyl) phthalate (MEOHP), and mono(2-ethylhexyl) phthalate (MEHP).

Overall, prepregnancy urinary phthalate metabolite and BPA concentrations did not change over time, except for MEP and MBzP concentrations (Table 3). On average, urinary MEP concentrations decreased 2% every 4 weeks (95% CI: –4, 0%) before conception. During pregnancy, concentrations of the sum of the four DEHP metabolites, the sum of the three oxidative DEHP metabolites, and the concentration of the hydrolytic monoester MEHP all decreased over time [Table 3; see Supplemental Material, Figure 2 (http://dx.doi.org/10.1289/ehp.1104139)]. The estimated average decreases in MEHP concentrations were larger than corresponding estimates for the sum of the three oxidative DEHP metabolites and the sum of all four DEHP metabolites. Decreases in urinary DEHP metabolite concentrations between the first and second trimester appeared to be responsible for this trend (see Supplemental Material, Table 1). Because our spaghetti plots [see Supplemental Material, Figure 2) suggested that urinary DEHP metabolites decreased between the first and second trimester and then rose between the second and third trimester, we included a time-squared polynomial term in our model. Concentrations decreased 54% (95% CI: –67, –37%) between the 5th and 20th week of gestation and increased 18% (95% CI: –13, 62%) between the 20th and 33rd week of gestation. Urinary concentrations of MBP, MBzP, and SG did not change over time during pregnancy, but MEP concentrations rose slightly during pregnancy (3%; 95% CI: –1, 7%) (Table 3).

Surrogate category analysis. In our classification analysis using the GM concentration of urinary phthalate metabolites as the assumed gold standard of gestational exposure, the top tertile of trimester-specific phthalate metabolite and BPA concentrations accurately classified the highest tertile of gestational exposure in at least 54% of women (Table 4). The PPVs varied across trimesters for phthalate metabolites and BPA. For instance, PPVs were highest for BPA concentrations in the first trimester (PPV = 0.70), but lowest in the second trimester (PPV = 0.60). Second- and third-trimester concentrations of MBP and MEP accurately classified ≥ 69% of women. Classification probabilities for the sum of DEHP metabolites and oxidative DEHP metabolites were similar to MEHP.

**Table 4:** Classification probabilities for the top tertile of average gestational BPA or phthalate concentration according to trimester-specific surrogate categories.^a,b^

Urinary metabolite	33rd percentile	66th percentile	Sensitivity	Specificity	PPVc
ΣDEHP (μmol/L)d										
First		0.22		0.89		0.69		0.84		0.69
Second		0.17		0.33		0.54		0.76		0.54
Third		0.15		0.45		0.62		0.80		0.62
MBP (μg/L)										
First		11		21		0.62		0.80		0.62
Second		12		23		0.73		0.86		0.73
Third		12		22		0.69		0.84		0.69
MBzP (μg/L)										
First		2.7		5.4		0.65		0.82		0.65
Second		2.3		5.1		0.62		0.80		0.62
Third		2.3		5.3		0.69		0.84		0.69
MEP (μg/L)										
First		20		73		0.62		0.80		0.62
Second		33		85		0.81		0.90		0.81
Third		28		81		0.77		0.88		0.77
BPA (μg/L)										
First		1.1		2.1		0.70		0.85		0.70
Second		1.0		1.9		0.60		0.80		0.60
Third		1.0		2.0		0.67		0.84		0.67
aAssumes that the within-woman GM gestational urinary phthalate or BPA concentration is the gold standard. The surrogate measure of low, medium, or high is defined by the first, second, or third tertile of trimester-specific urine samples, respectively. Probabilities are calculated from the top versus bottom two tertiles of trimester-specific and average gestation urinary phthalate metabolite and BPA concentrations. Limited to women with all three urine samples. All concentrations are SG adjusted. bn = 77 for phthalates; n = 91 for BPA. cPPV is the probability of being classified in the top tertile of mean gestational phthalate metabolite or BPA concentration, given that the woman’s trimester-specific urinary phthalate metabolite or BPA concentration is in the top tertile. dΣDEHP metabolites: mono 2-ethyl-5-carboxypentyl phthalate (MECPP), mono 2-ethyl-5-hydroxyhexyl phthalate (MEHHP), mono 2-ethyl-5-oxohexyl phthalate (MEOHP), and mono 2-ethylhexyl phthalate (MEHP).										

We observed that average gestational urinary phthalate metabolite/BPA concentrations increased across tertiles of trimester-specific concentrations (Figure 1). Average gestational phthalate metabolite/BPA concentrations were lowest among participants with trimester-specific samples classified in the lowest tertile and highest among women in the top tertiles. However, the range of average gestational concentrations between adjacent surrogate categories overlapped in at least one trimester for each phthalate metabolite and BPA.

In our final surrogate analysis, at least 77% of women had two or more urine samples in the same tertile of urinary phthalate metabolite or BPA concentrations during the course of pregnancy (Table 5). The proportion of women with all three urine samples in the same category during each trimester ranged from 16% (for DEHP) to 26% (for MBP).

**Table 5:** Number (%) of women with individual spot urine samples in the same tertile of exposure during pregnancy.^a^

No. of samples in the same tertile	ΣDEHP metabolitesb	MBP	MBzP	MEP	BPA
None		16 (21)		18 (23)		16 (21)		16 (21)		16 (18)
2		49 (64)		39 (51)		49 (64)		48 (62)		54 (59)
3		12 (16)		20 (26)		12 (16)		13 (17)		21 (23)
aLimited to women with all three pregnancy urine samples (n = 77 for phthalate analysis; n = 91 for BPA analysis). bDEHP metabolites: mono(2-ethyl-5-carboxypentyl) phthalate (MECPP), mono(2-ethyl-5-hydroxyhexyl) phthalate (MEHHP), mono(2-ethyl-5-oxohexyl) phthalate (MEOHP), and mono(2-ethylhexyl) phthalate (MEHP).										

## Discussion

The absolute differences in urinary concentrations of DEHP metabolites, MBP, MiBP, MBzP, and BPA were relatively small before and during pregnancy, considering the variability of our analytic chemistry methods. Urinary concentrations of MBP, MBzP, and MEP were modestly correlated before and during pregnancy, whereas DEHP metabolites and BPA were less correlated during the two time periods. Serial urine concentrations of BPA and some phthalate metabolites were highly variable before and during pregnancy. The variability of most urinary phthalate metabolites was similar before and during pregnancy, whereas the variability of BPA and MBzP increased during pregnancy. During pregnancy, urinary concentrations of DEHP metabolites decreased and were the most variable. MBP and MEP concentrations were less variable, and MEP and MiBP concentrations increased during pregnancy.

The variability of urinary phthalate metabolite and BPA concentrations in our study participants was remarkably similar to that of most previous studies that collected multiple urine samples over weeks to months from the same individuals (Adibi et al. 2008; Baird et al. 2010; Braun et al. 2011; Hauser et al. 2004; Irvin et al. 2010; Peck et al. 2010). Previous studies of phthalate metabolites have all shown a similar pattern where the concentration of MEHP is the most variable and concentrations of MBP, MEP, and MBzP are less variable. MEP concentrations (ICC = 0.21) among pregnant women from New York City were more variable than MEP concentrations in our study participants (Adibi et al. 2008). Source population characteristics and variations in exposure sources could influence within-woman variability across cohorts.

To our knowledge, no studies have examined whether phthalate metabolite and BPA concentrations or their within-person variability are different before or during pregnancy in the same woman. Differences would suggest that physiological changes during pregnancy may influence the absorption, distribution, metabolism, or excretion of phthalate metabolites and BPA. The absolute differences in urinary concentrations of phthalate metabolites and BPA were relatively small, suggesting that women did not change their behaviors to reduce these exposures. We did not observe a consistent increase or decrease in most phthalate metabolites or BPA over the course of pregnancy, which suggests that urinary concentrations of these compounds are not impacted by pregnancy-induced changes in pharmacokinetics. However, urinary concentrations of DEHP metabolites decreased during pregnancy. Behavioral changes may be responsible for the decrease in urinary DEHP metabolite concentrations between the first and second trimesters of pregnancy, because women may have begun eating more meals consisting of fresh foods with less packaging after learning of their pregnancy (Rudel et al. 2011).

The different variability among urinary phthalate metabolite and BPA concentrations may be attributable to their exposure sources. Diet is believed to be the main source of DEHP and BPA exposure, primarily from PVC materials used in the processing/storage of food and polycarbonate food/beverage containers, respectively (Chapin et al. 2008; Lopez-Cervantes and Paseiro-Losada 2003; Petersen and Jensen 2010; von Goetz et al. 2010). Other sources of BPA exposure may include contact with thermal receipts (Braun et al. 2011; Liao and Kannan 2011). Higher day-to-day and within-day variability in dietary sources is likely responsible for the higher variability of urinary DEHP metabolites and BPA concentrations relative to MEP and MBP concentrations, whose primary sources include personal care and beauty products (Preau et al. 2010; Ye et al. 2011). Although the half-lives of the DEHP metabolites vary, we observed similar variability of both MEHP and MECPP, suggesting that the sources of exposure may be more important than half-life of the metabolite.

MEP and MBP concentrations were relatively stable and reproducible in repeated samples collected before and during pregnancy. Preau et al. (2010) reported that urinary MEP concentrations among eight adult volunteers were variable during the day but exhibited similar patterns across days within the same individual. One possible explanation may be that people use the same DBP- and DEP-containing personal care or cosmetic products from day to day and at similar times during the day, which may be responsible for the reduced variability in DBP and DEP urinary metabolites relative to other phthalate metabolites (Duty et al. 2005; Romero-Franco et al. 2011). Furthermore, phthalates found in personal care products will be absorbed across the skin over a longer period of time and bypass first-pass metabolism in the liver, which might increase their apparent half-life and reduce the variability of their metabolite concentrations in urine within a given day.

Our results suggest that MEP and MBP concentrations measured in a single spot urine sample in the second or third trimester of pregnancy might reasonably classify gestational exposure to DEP and DBP, respectively. However, reproducibility was substantially lower in samples collected in the first trimester. Given that DEHP metabolites, MBzP, and BPA were more variable, more than one sample may be necessary to adequately classify gestational exposure to these compounds. Most sampling strategies will not completely eliminate all variability and will still result in some exposure misclassification.

Our surrogate category analysis has at least two limitations. First, we assumed that GM phthalate metabolite and BPA concentrations from three urine samples during pregnancy were indicative of exposure during the entire gestation. However, it is likely that there were additional exposures and sources of exposure variability that we were unable to capture. Serial urine collections will not be possible in every epidemiological study, and researchers are encouraged to conduct their own exposure validation when possible. Second, our surrogate analyses may overestimate the accuracy of a single spot urine sample because of the nonindependence of the surrogate and average measures. Other studies with more than three urine samples during pregnancy are needed to determine whether a single spot urine sample, not included in the calculation of average exposure, can accurately classify gestational exposure.

The primary strength of this study was the availability of multiple urine samples obtained both before and during pregnancy from the same woman. Furthermore, we collected urine samples very early in pregnancy, allowing us to examine exposures and exposure variability across the entire pregnancy. However, we were not able to examine BPA and phthalate metabolite variability within specific trimesters, which may be important in identifying critical windows of exposure during pregnancy. Depending on the end point of interest, it may be more relevant to classify exposure during a narrow window of gestation early in pregnancy instead of during the entire period of gestation (Ge et al. 2007). Although we were unable to estimate variability within narrower windows of gestation, our findings may be used to guide and evaluate studies examining gestational phthalate/BPA exposures and maternal/child health outcomes. Given the consistent magnitude of phthalate metabolite/BPA variability observed in previous studies conducted over time spans ranging from days to months, we believe the variability within trimesters or narrower windows would be similar to the variability over the course of pregnancy, especially considering the short half-life and nonpersistent nature of phthalates and BPA (Adibi et al. 2008; Baird et al. 2010; Braun et al. 2011; Hauser et al. 2004; Irvin et al. 2010; Peck et al. 2010; Preau et al. 2010; Ye et al. 2011).

Source population characteristics could influence phthalate/BPA exposures and their absorption, distribution, metabolism, and excretion. Therefore, these results may not be generalizable to other populations. Women in this study were from higher socioeconomic position and were aware that this study was examining the health effects of phthalates and other environmental chemicals. These factors might increase the likelihood of behavioral changes before and during pregnancy that would not be observed in other source populations. In addition, these women were older than women from studies among couples conceiving naturally and were partners in couples seeking treatment for infertility. Physiological changes associated with advanced age or subfertility may be responsible for some of the observed results in this cohort.

In conclusion, urinary phthalate metabolite and BPA concentrations were variable before and during pregnancy in this cohort. MEP and MBP concentrations were less variable and more correlated before and during pregnancy than DEHP metabolites, MBzP, and BPA. Our findings suggest that a single spot urine sample may permit relatively accurate classification of DBP and DEP exposure during pregnancy, but accurate classification of DEHP, BzBP, and BPA exposure may require multiple urine samples. Our variability and surrogate category estimates can be used to assess the extent to which phthalate and BPA exposure misclassification may bias results in epidemiological studies. Future studies should investigate the relative contribution of pharmacokinetic and behavioral factors to urinary phthalate metabolite and BPA concentration variability.
