# Erratum: Braun et al. “Variability of Urinary Phthalate Metabolite and Bisphenol A Concentrations before and during Pregnancy” [120:739–745 (2012)]

**DOI:** 10.1289/ehp.121-a114

**Published:** 2013-04-01

Braun et al. discovered an error in the SAS code of their paper “Variability of Urinary Phthalate Metabolite and Bisphenol A Concentrations before and during Pregnancy” [Environ Health Perspect 120:739–745 (2012)]. The error resulted in the reported absolute concentrations of phthalate metabolites and BPA being approximately 50% higher. The authors mistakenly used the median value from the EARTH study men [specific gravity (SG) = 1.025] rather than women (SG = 1.015) in their SG calculations. Thus, the reported concentrations in Table 2 (columns 2 and 3), Table 4 (columns 2 and 3), and Figure 1, as well as in the Supplemental Material, were higher than if the median SG for this group of women had been used. The authors note that this error did not alter the statistical analyses or their interpretations or the conclusions of their paper.

**Table 2:** Univariate characteristics, correlations, and difference between SG-adjusted prepregnancy and pregnancy urinary phthalate metabolite and BPA concentrations.

Urine metabolite	GM prepregnancy median (25th, 75th percentile)a	GM pregnancy median (25th, 75th percentile)a	Correlationb	Percent difference between pregnancy and prepregnancy concentration (95% CI)c
ΣDEHP metabolitesd (μmol/L)	0.33 (0.20, 0.56)	0.36 (0.18, 0.69)	0.33	9 (–8, 29)
ΣDEHP oxidative metabolitese (μmol/L)	0.30 (0.18, 0.52)	0.31 (0.17, 0.66)	0.34	8 (–9, 28)
MEHP (μg/L)	5.0 (2.8, 7.7)	5.9 (3.2, 13)	0.32	29 (8, 55)
MBP (μg/L)	14 (9.0, 22)	16 (11, 24)	0.62	25 (11, 41)
MiBP (μg/L)	5.4 (3.2, 8.9)	5.9 (4.0, 9.0)	0.55	9 (–4, 23)
MBzP (μg/L)f	3.2 (1.8, 5.2)	3.7 (2.0, 6.8)	0.54	22 (6, 40)
MEP (μg/L)f	61 (37, 145)	55 (24, 116)	0.62	–19 (–30, –6)
BPA (μg/L)	1.5 (1.1, 2.1)	1.5 (1.1, 2.3)	0.39	6 (–4, 17)
aMedian and percentiles of the within-woman GM of urinary phthalate metabolite or BPA concentrations in two or more samples obtained before pregnancy and during pregnancy. bPearson correlation coefficients between the GM of prepregnancy and pregnancy urinary phthalate metabolite or BPA concentrations (log10 transformed). cPercent difference in pregnancy versus prepregnancy urinary phthalate metabolite or BPA concentration; estimated with a linear mixed model with an indicator variable for pregnancy or prepregnancy (referent). dΣDEHP metabolites: mono(2-ethyl-5-carboxypentyl) phthalate (MECPP), mono(2-ethyl-5-hydroxyhexyl) phthalate (MEHHP), mono(2-ethyl-5-oxohexyl) phthalate (MEOHP), and mono(2-ethylhexyl) phthalate (MEHP). eΣDEHP oxidative metabolites: mono(2-ethyl-5-carboxypentyl) phthalate (MECPP), mono(2-ethyl-5-hydroxyhexyl) phthalate (MEHHP), and mono(2-ethyl-5-oxohexyl) phthalate (MEOHP). fA correction factor of 0.66 and 0.72 was applied to the MEP and MBzP concentrations, respectively.								

**Table 4:** Classification probabilities for the top tertile of average gestational BPA or phthalate concentration according to trimester-specific surrogate categories.^*a,b*^

Urinary metabolite	33rd percentile	66th percentile	Sensitivity	Specificity	PPVc
ΣDEHP (μmol/L)d										
First	0.22	0.89	0.69	0.84	0.69
Second	0.17	0.33	0.54	0.76	0.54
Third	0.15	0.45	0.62	0.80	0.62
MBP (μg/L)
First	11	21	0.62	0.80	0.62
Second	12	23	0.73	0.86	0.73
Third	12	22	0.69	0.84	0.69
MBzP (μg/L)
First	2.7	5.4	0.65	0.82	0.65
Second	2.3	5.1	0.62	0.80	0.62
Third	2.3	5.3	0.69	0.84	0.69
MEP (μg/L)
First	20	73	0.62	0.80	0.62
Second	33	85	0.81	0.90	0.81
Third	28	81	0.77	0.88	0.77
BPA (μg/L)
First	1.1	2.1	0.70	0.85	0.70
Second	1.0	1.9	0.60	0.80	0.60
Third	1.0	2.0	0.67	0.84	0.67
aAssumes that the within-woman GM gestational urinary phthalate or BPA concentration is the gold standard. The surrogate measure of low, medium, or high is defined by the first, second, or third tertile of trimester-specific urine samples, respectively. Probabilities are calculated from the top versus bottom two tertiles of trimester-specific and average gestation urinary phthalate metabolite and BPA concentrations. Limited to women with all three urine samples. All concentrations are SG adjusted. bn = 77 for phthalates; n = 91 for BPA. cPPV is the probability of being classified in the top tertile of mean gestational phthalate metabolite or BPA concentration, given that the woman’s trimester-specific urinary phthalate metabolite or BPA concentration is in the top tertile. dΣDEHP metabolites: mono 2-ethyl-5-carboxypentyl phthalate (MECPP), mono 2-ethyl-5-hydroxyhexyl phthalate (MEHHP), mono 2-ethyl-5-oxohexyl phthalate (MEOHP), and mono 2-ethylhexyl phthalate (MEHP).										

**Figure:**
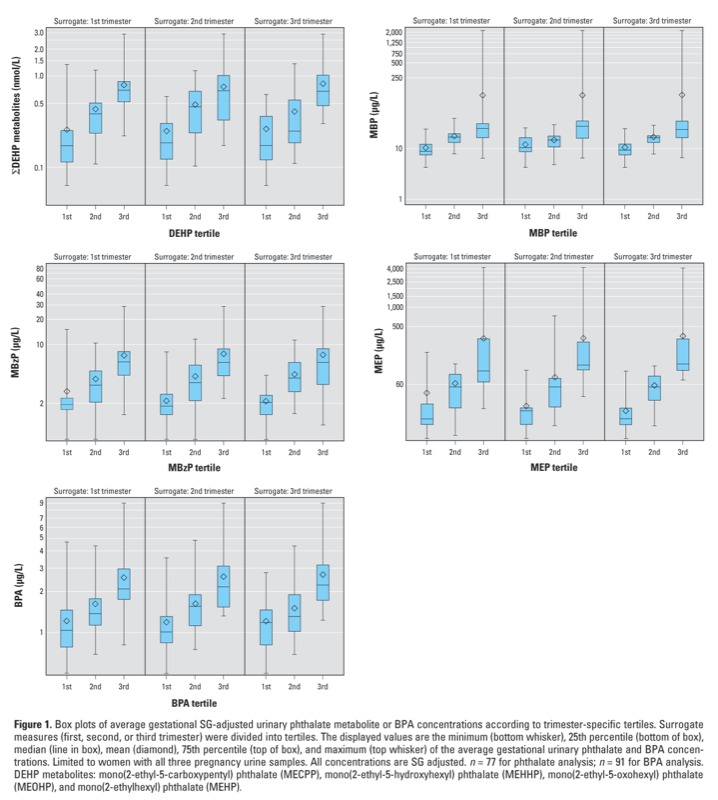
Box plots of average gestational SG-adjusted urinary phthalate metabolite or BPA concentrations according to trimester-specific tertiles. Surrogate measures (first, second, or third trimester) were divided into tertiles. The displayed values are the minimum (bottom whisker), 25th percentile (bottom of box), median (line in box), mean (diamond), 75th percentile (top of box), and maximum (top whisker) of the average gestational urinary phthalate and BPA concentrations. Limited to women with all three pregnancy urine samples. All concentrations are SG adjusted. *n* = 77 for phthalate analysis; *n* = 91 for BPA analysis. DEHP metabolites: mono(2-ethyl-5-carboxypentyl) phthalate (MECPP), mono(2-ethyl-5-hydroxyhexyl) phthalate (MEHHP), mono(2-ethyl-5-oxohexyl) phthalate (MEOHP), and mono(2-ethylhexyl) phthalate (MEHP).

The authors regret the errors.

